# Multi-Layered Unsupervised Learning Driven by Signal-to-Noise Ratio-Based Relaying for Vehicular Ad Hoc Network-Supported Intelligent Transport System in eHealth Monitoring

**DOI:** 10.3390/s24206548

**Published:** 2024-10-11

**Authors:** Ali Nauman, Adeel Iqbal, Tahir Khurshaid, Sung Won Kim

**Affiliations:** 1 School of Computer Science and Engineering, Yeungnam University, Gyeongsan-si 38541, Republic of Korea; anauman@ynu.ac.kr (A.N.); adeeliqbal@yu.ac.kr (A.I.); swon@yu.ac.kr (S.W.K.); 2 Department of Electrical Engineering, Yeungnam University, Gyeongsan-si 38541, Republic of Korea

**Keywords:** unsupervised learning, Internet of Things (IoT), Intelligent Transportation System (ITS), relaying, vehicular ad hoc network (VANET), eHealth, continuous health monitoring, real-time data processing

## Abstract

Every year, about 1.19 million people are killed in traffic accidents; hence, the United Nations has a goal of halving the number of road traffic deaths and injuries by 2030. In line with this objective, technological innovations in telecommunication, particularly brought about by the rise of 5G networks, have contributed to the development of modern Vehicle-to-Everything (V2X) systems for communication. A New Radio V2X (NR-V2X) was introduced in the latest Third Generation Partnership Project (3GPP) releases which allows user devices to exchange information without relying on roadside infrastructures. This, together with Massive Machine Type Communication (mMTC) and Ultra-Reliable Low Latency Communication (URLLC), has led to the significantly increased reliability, coverage, and efficiency of vehicular communication networks. The use of artificial intelligence (AI), especially K-means clustering, has been very promising in terms of supporting efficient data exchange in vehicular ad hoc networks (VANETs). K-means is an unsupervised machine learning (ML) technique that groups vehicles located near each other geographically so that they can communicate with one another directly within these clusters while also allowing for inter-cluster communication via cluster heads. This paper proposes a multi-layered VANET-enabled Intelligent Transportation System (ITS) framework powered by unsupervised learning to optimize communication efficiency, scalability, and reliability. By leveraging AI in VANET solutions, the proposed framework aims to address road safety challenges and contribute to global efforts to meet the United Nations’ 2030 target. Additionally, this framework’s robust communication and data processing capabilities can be extended to eHealth monitoring systems, enabling real-time health data transmission and processing for continuous patient monitoring and timely medical interventions. This paper’s contributions include exploring AI-driven approaches for enhanced data interaction, improved safety in VANET-based ITS environments, and potential applications in eHealth monitoring.

## 1. Introduction

Approximately 1.19 million lives are lost every year due to road traffic accidents, making injuries the most common cause of death among children and young adults aged 5–29 [1]. Although they own only 60% of the world’s vehicles, low and middle-income countries account for a staggering 92% of road traffic fatalities. More than half of these deaths involve vulnerable road users such as pedestrians, cyclists, and motorcyclists. The economic burden is significant since this kind of accident accounts for approximately 3% of a nation’s GDP [2]. The United Nations General Assembly has established a goal to reduce road traffic deaths and injuries globally by half by 2030 as part of this global crisis management plan [3,4].

As developments in telecommunication technologies have occurred, especially with the widespread integration of wireless communication systems, there has been a growing focus on the integration of sensors with wireless antennas that have the capacity to convey data over considerable distances [5,6,7,8,9]. This synthesis has facilitated the acquisition of vital information from inaccessible regions, thereby establishing a foundation for improved communication protocols and safety initiatives. In the contemporary milieu of fifth-generation (5G) mobile communication and its subsequent developments, the advent of massive machine-type communication (mMTC) and ultra-reliable low-latency communication (URLLC) has markedly increased the connectivity and dependability of Internet of Things (IoT) devices, thereby reinforcing communication frameworks across various sectors, including transportation and healthcare [10,11,12,13].

The advent of 5G networks has facilitated the introduction of a multitude of innovative features pertinent to Vehicle-to-Everything (V2X) communication  [14,15]. The releases from the 3rd Generation Partnership Project (3GPP), specifically Release 16 and Release 17, have inaugurated the New Radio V2X (NR-V2X) standard [14], which encompasses SideLink (SL) communication. This technological advancement permits direct radio communication between devices, including vehicles and personal electronic devices, eliminating the need for roadside units to facilitate data transfer. This progress markedly enhances vehicular ad hoc network (VANET) communication, addressing the escalating requirements for safety, commercial applications, and infotainment services [16]. Furthermore, ongoing initiatives by the 3GPP are directed towards improving communication reliability, extending coverage, minimizing latency, and optimizing power consumption, particularly for devices reliant on battery power [17,18,19,20].

VANETs, a distinct category of mobile ad hoc networks (MANETs), are integral to the functionality of Intelligent Transportation Systems (ITSs) [21,22,23,24]. VANETs facilitate communication between vehicular units and adjacent roadside infrastructure, wherein vehicles serve as mobile nodes within a self-organizing network framework. These networks operate without the necessity for prior coordination, as each vehicle operates as a wireless routing device, thereby enabling interaction with proximate vehicles. In metropolitan contexts, this communicative exchange generally transpires within a radius of 100 to 300 m, with potential extension up to 1000 m on arterial highways [17,18]. As vehicles transition in and out of communication range, the network undergoes continuous adaptation and reconfiguration, culminating in a fluid and dynamic communicative milieu [19].

Recently, the utilization of artificial intelligence (AI) has increased across diverse domains, encompassing cybersecurity, data analytics, routing, healthcare, and robotics [25,26,27]. AI methodologies, including machine learning (ML), deep learning (DL), and swarm intelligence (SI), are increasingly being recognized in the formulation of solutions for vehicular ad hoc networks (VANETs) to mitigate their myriad challenges [28,29]. Although AI has demonstrated considerable potential in enhancing vehicular communication, additional investigation is imperative to thoroughly harness its capabilities within this sector.

A prominent AI methodology that has demonstrated substantial potential within VANETs is K-means clustering. This specific type of unsupervised machine learning technique, which is part of the larger domain of artificial intelligence, is particularly effective in facilitating efficient data exchange among vehicles [30,31,32,33,34,35]. Through the implementation of a clustering-oriented topology, K-means categorizes vehicles situated in close geographical proximity, thereby facilitating direct communication within a cluster and inter-cluster data transmission via designated cluster heads. This strategy significantly augments both the scalability and dependability of data exchange within VANETs.

Given the promising role of AI, particularly K-means clustering, in improving VANET performance, this paper proposes a multi-layered VANET-enabled ITS framework, driven by unsupervised learning. This framework is designed to address the key challenges in VANET communication and help meet the United Nations’ goal of reducing road traffic fatalities by 50% by 2030. Table 1 provides a summary of the literature on road traffic safety, VANETs, and AI applications with identified research gaps.

The contributions of this paper are as follows:

1. Introduction of a novel multi-layered, AI-powered VANET framework for ITS.
2. Exploration of K-means clustering for optimized data interaction among vehicles.
3. Application of unsupervised learning to enhance VANET scalability and reliability.

### Organization

The structure of this paper is as follows: The system model is described in detail in Section 2, including its components and architecture. The methodology and approach used to tackle the challenges faced in VANET communication are outlined in Section 3 as a proposed solution. In Section 4, simulation results are provided, showing how effectively the solution fares in different scenarios in terms of performance. Finally, Section 5 gives an overall conclusion that summarizes the main findings and contributions of the research work.

## 2. System Model

**Figure 1:**
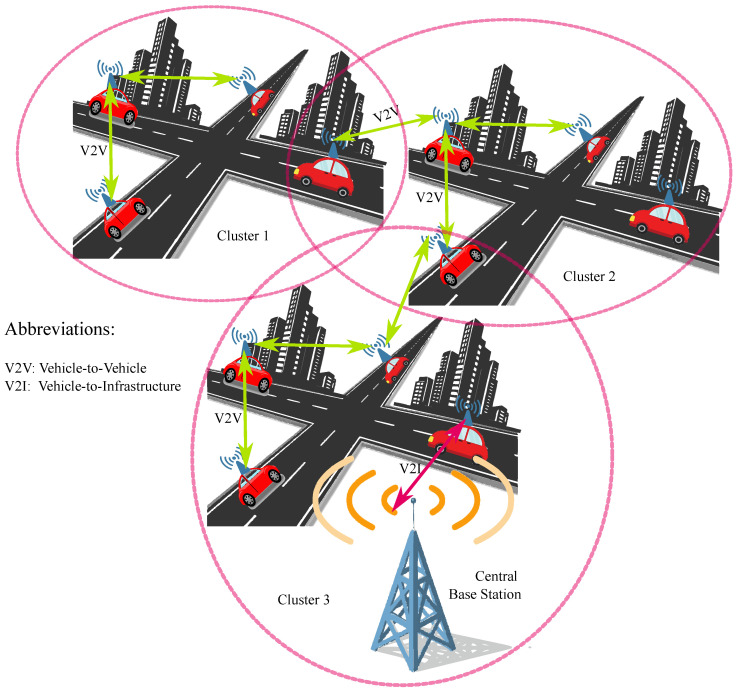
An illustration of the multi-layered unsupervised learning-based and VANET-enabled ITS framework.

In this section, a conceptual framework for a VANET-enhanced ITS is developed, emphasizing the interactions between Vehicle-to-Vehicle (V2V) and Vehicle-to-Infrastructure (V2I) communications. The framework posits a network of automobiles, each of which possesses the ability to engage in indirect communication with fellow vehicles alongside roadside units (RSUs), thereby establishing a fluid and self-regulating network. The principal aim of the system is to increase safety and optimize traffic management through the proficient exchange and analysis of data within VANETs. Figure 1 shows the overall network topology, where vehicles operate in a cooperative manner to ensure real-time data sharing, which is crucial for maintaining road safety and traffic efficiency.

### 2.1. K-Means Clustering Algorithm

At the foundation of this framework lies the implementation of the K-means clustering algorithm, an extensively utilized unsupervised learning methodology for the categorization of data points predicated on their similarity. Within the framework of VANETs, K-means clustering is employed to categorize vehicles into distinct clusters according to their spatial proximity, Signal-to-Noise Ratio (SNR) metrics, and prevailing traffic patterns. This clustering mechanism facilitates the efficient exchange of data within the clusters and enhances inter-cluster communication by designating cluster heads tasked with the responsibility of transmitting information.

Each vehicle is assumed to periodically calculate its SNR values with respect to nearby vehicles. Based on these values, the K-means algorithm groups vehicles into clusters, optimizing communication paths and improving data transmission reliability.

The clustering process is mathematically modeled as follows:

Let $V=\{v_{1},v_{2},\ldots ,v_{n}\}$ represent the set of vehicles in the network, and let $P=\{p_{1},p_{2},\ldots ,p_{k}\}$ represent the set of cluster centroids, where *k* is the number of clusters. The objective of the K-means algorithm is to minimize the intra-cluster variance, defined as:

$$J=\sum_{i=1}^{k}\sum_{v_{j}\in C_{i}}{∥v_{j}-p_{i}∥}^{2}$$

where $C_{i}$ represents the set of vehicles assigned to cluster *i*, and $∥v_{j}-p_{i}∥$ denotes the Euclidean distance between vehicle $v_{j}$ and the centroid $p_{i}$.

The centroids are updated iteratively as follows:

$$p_{i}=\frac{1}{|C_{i}|}\sum_{v_{j}\in C_{i}}v_{j}$$

The clustering process continues until the centroids converge, i.e., there is no significant change in their positions across iterations.

### 2.2. Collision Detection and Alert System

In addition to clustering, the system continuously analyzes vehicle data to detect potential collision risks. This is achieved by comparing the relative speeds and positions of vehicles within each cluster. If the system determines that two or more vehicles are on a likely collision path, based on their proximity and velocity, it triggers an immediate alert. This alert is shared with all vehicles in the cluster to take preventive measures, such as slowing down or changing lanes.

Mathematically, the system assesses collision risk based on the predicted future positions of vehicles. For two vehicles $v_{i}$ and $v_{j}$, their future positions after time *t* are calculated as:

$$P_{i}\left(t\right)=P_{i}\left(0\right)+v_{i}\cdot t$$

$$P_{j}\left(t\right)=P_{j}\left(0\right)+v_{j}\cdot t$$

where $P_{i}\left(0\right)$ and $P_{j}\left(0\right)$ are the current positions of the vehicles, and $v_{i}$ and $v_{j}$ are their respective velocities. If the predicted distance between the two vehicles at time *t* falls below a predefined safety threshold $d_{\mathrm{threshold}}$, a collision alert is generated:

$$∥P_{i}\left(t\right)-P_{j}\left(t\right)∥<d_{\mathrm{threshold}}$$

### 2.3. Information Relay and Dissemination

Once a collision alert is generated, it is critical that this information is transmitted to nearby vehicles and relevant authorities in a timely manner. The proposed system employs a tier-2 algorithm to relay this information across the VANET. Vehicles located at the edge of a cluster, based on their SNR values, act as relays to forward the alert to other clusters and the central base station.

The SNR for each vehicle is calculated as:

$${\mathrm{SNR}}_{i}=\frac{P_{\mathrm{signal}}}{P_{\mathrm{noise}}}$$

where $P_{\mathrm{signal}}$ and $P_{\mathrm{noise}}$ represent the signal power and noise power, respectively.

### 2.4. Traffic Management and Data Analysis

The data collected through the VANET are not only used for immediate safety measures but also contribute to long-term traffic management. The central base station collects traffic data from multiple clusters and applies the K-means algorithm to identify congestion patterns within different regions. By clustering vehicles based on their density and speed, the system can locate areas with high traffic density and predict potential congestion hotspots.

## 3. Proposed Solution

This segment delineates the suggested triadic, multi-tiered resolution, wherein the resultant output of each phase functions as the input for the ensuing phase. Each phase is elaborated upon through relevant algorithms.

### 3.1. Stage 1

Algorithm 1 emulates the operational behavior of a vehicle within a VANET, emphasizing the significance of real-time data transmission, collision detection, and surveillance of vehicle trajectories. It establishes essential parameters, including vehicle velocity, geographic location, and directional orientation, in addition to parameters pertinent to K-means clustering, which facilitates the identification of potential collisions predicated on the spatial proximity of adjacent vehicles. During each computational iteration, the vehicle’s positional coordinates and speed are recalibrated, data are disseminated to RSUs or proximal vehicles, and the amalgamated data are subjected to clustering techniques to ascertain collision hazards. In instances where a collision is anticipated, notifications are generated for both the driver and surrounding vehicles. Furthermore, the vehicle’s velocity and trajectory are continuously scrutinized, and driving-related data are relayed to centralized entities, including insurance providers and traffic regulatory authorities.
**Algorithm 1** An AI-enabled VANET-based collision detection system1:Initialize vehicle parameters: ID, speed, location, direction, and nearby vehicles.2:Set K-means parameters for collision detection.3:**while** vehicle is running **do**4:   Update vehicle’s speed, location, and direction.5:   Transmit data to nearby vehicles and RSUs.6:   Receive and combine data from nearby vehicles.7:   Perform K-means clustering on vehicle locations.8:   Check if any cluster is within collision threshold.9:   **if** collision detected **then**10:     Alert driver and notify nearby vehicles.11:   **end if**12:   Send data to central systems.13:   Simulate vehicle running status and pause.14:**end while**

### 3.2. Stage 2

Algorithm 2 utilizes the results obtained from Algorithm 1 and implements K-means clustering predicated on SNR to categorize the vehicles into distinct clusters. Subsequent to the clustering process, the peripheral vehicles within each cluster are discerned as those situated at the greatest distance from the cluster centroids. These peripheral vehicles are subsequently assigned the responsibility of relaying information to proximate vehicles within a designated distance threshold. The algorithm culminates by delineating the positions of the vehicles, identifying the peripheral vehicles, and establishing the communication pathways to facilitate the visualization of the network and the flow of information.
**Algorithm 2** SNR-based information spreading using K-means clustering1:**Step 1:** Input data from Algorithm 1.2:**Step 1:** Calculate SNR based on distance.3:**Step 2:** Apply K-means clustering on vehicles.4:**Step 3:** Identify edge vehicles in each cluster by finding the vehicle farthest from the cluster centroid.5:**Step 4:** Transmit information from edge vehicles to nearby vehicles within a defined distance threshold.6:Identify vehicle positions, clusters, edge vehicles, and transmission lines.

### 3.3. Stage 3

Algorithm 3 operationalizes a real-time intelligent traffic management framework that incessantly acquires traffic data from Algorithms 1 and 2, respectively. Employing K-means clustering, it delineates areas of high density to ascertain congestion. Upon the identification of congestion, the system autonomously modifies traffic signal timings, redirects vehicles, and disseminates notifications to proximate vehicles. In the occurrence of an incident, emergency services are alerted, traffic is rerouted, and the duration of green lights is prolonged for the routes affected. In the absence of both congestion and incidents, the algorithm enhances the optimization of traffic signals to facilitate a seamless flow.
**Algorithm 3** Intelligent traffic management system1:Input data from Algorithms 1 and 2.2:**while** system is running **do**3:   Collect and analyze traffic data using K-means clustering.4:   Check for congestion or incidents.5:   **if** congestion detected **then**6:     Adjust signals, reroute traffic, and notify vehicles.7:   **else if** incident detected **then**8:     Notify emergency services, divert traffic, and adjust signals.9:   **else**10:     Optimize traffic signals for smooth flow.11:   **end if**12:   Update traffic management strategies.13:   Wait for the next update interval.14:**end while**

### 3.4. Computational Complexity

The proposed framework consists of three stages, and the computational complexity of each stage is as follows:

- For stage 1, the worst-case computational complexity is O(t×i), where *i* is the number of iterations for K-means clustering and *t* is the expected number of iterations of the main loop.
- For stage 2, the worst-case computational complexity is O(N×C×i), where *i* is the number of iterations for K-means clustering, *N* is the number of vehicles, and *C* is the number of clusters.
- Similarly, stage 3 is also dominated by k-means clustering, with the worst-case computational complexity of O(N×C×i). In stage 3, the value of *N* is larger than that of stage 2.

The above three-stage framework provides a near-optimal solution. In order to find the optimal solution, we have to adopt the exhaustive-search-based method. In solving the above algorithm using exhaustive search, the worst-case computational complexity is $O(C^{N})$, which is exponentially more expensive than K-means clustering, making it infeasible for large datasets.

## 4. Results

In this section, the functionality of the proposed multi-tiered solution is rigorously substantiated through comprehensive MATLAB-based simulation analysis, alongside the integration of real-time simulation parameters.

**Figure 2:**
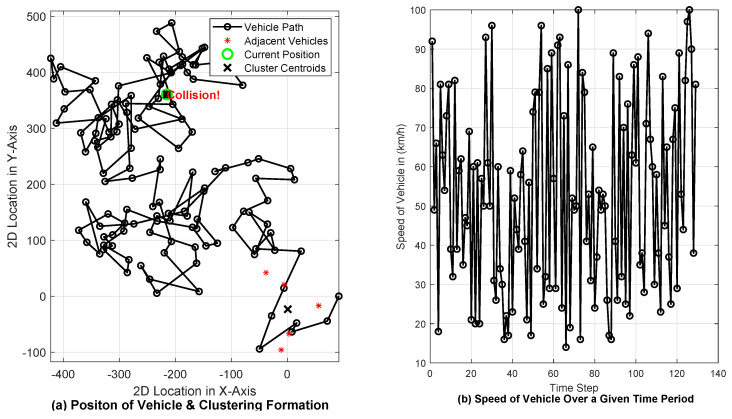
Analysis of the proposed collision detection algorithm, where (**a**) illustrates the vehicle path and detects collisions and (**b**) illustrates the variation in vehicle speed over a given time period.

In Figure 2, a real time simulation of a V2X communication system in a VANET is demonstrated using real-time data transmission, clustering for collision detection, and traffic management strategies. The velocity of each vehicle fluctuates within the range of 10 km/h to 100 km/h, with each vehicle designated a distinct identifier commencing from 1 and concluding at 100, as it has been posited that a total of 100 vehicles are utilized to model the proposed mechanism, wherein the communication range of each vehicle is established at 100 m. Finally, the wireless channel parameter is based on [36]. Figure 2a visualizes the vehicle’s trajectory, nearby vehicles, and cluster centroids. The K-means clustering algorithm identifies these centroids, which are used to determine high-risk zones for potential collisions. When the vehicle’s location falls within a critical distance of a centroid, a collision alert is triggered, demonstrating the system’s ability to detect and respond to collision risks in real time. Additionally, the vehicle notifies nearby vehicles, showcasing effective V2V communication. Figure 2b shows the vehicle’s speed over time, providing insight into the speed fluctuations during the simulation. This reflects how speed changes dynamically throughout the scenario, which is relevant for evaluating the real-time performance of the system. The simulation also includes continuous data transmission to a central traffic system and insurance companies, ensuring that driving data are logged and shared for further analysis and decision-making. Overall, the results showcase the system’s ability to manage vehicle trajectories, detect potential collisions, and optimize communication between vehicles and infrastructure, thereby contributing to enhanced road safety and traffic efficiency in a VANET environment.

**Figure 3:**
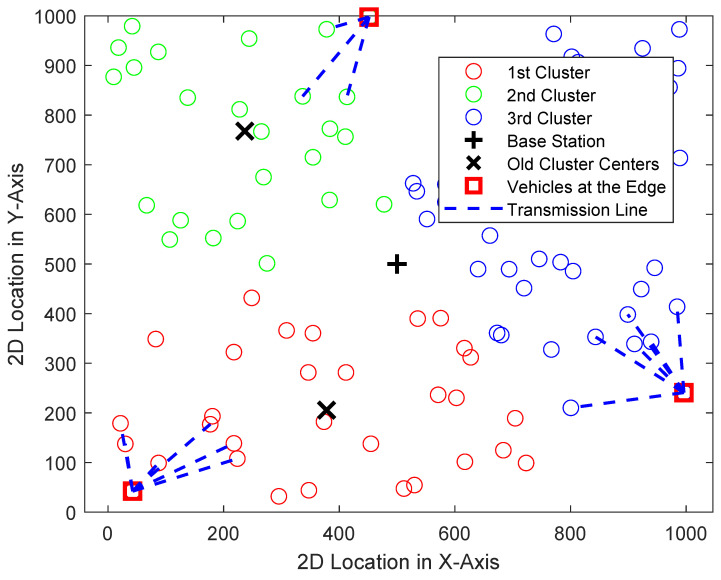
Analysis of proposed SNR-based information spread system, where results identify the edge vehicles and new information path.

Figure 3 shows the results of the simulation, which modeled a VANET scenario with 100 vehicles randomly positioned within a 1000 × 1000 unit area, using the outputs of Algorithm 1. Using the K-means clustering algorithm, these vehicles were grouped into three distinct clusters based on their geographic locations. The base station, positioned at the center of the area, served as a reference for calculating SNR values. Additionally, edge vehicles—those furthest from their cluster centroids—were identified. These edge vehicles play a key role in the communication process by relaying information to nearby vehicles within their cluster, ensuring data dissemination across the network. The simulation shows these communication paths through blue dashed lines, illustrating how edge vehicles transmit data to neighboring nodes within their respective clusters. These results highlight the effectiveness of K-means clustering in organizing vehicles into groups based on proximity, facilitating efficient communication between vehicles within each cluster and extending the range of information dissemination through edge vehicles.

**Figure 4:**
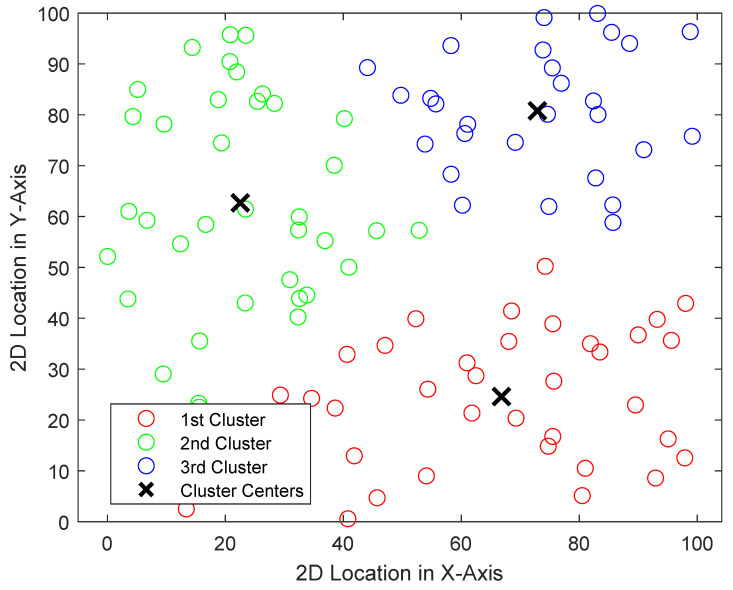
An illustration of the multilayered, unsupervised, learning-based, and VANET-enabled ITS framework.

In Figure 4, the results of the traffic data analysis are presented through a visual representation of a real-time vehicular network using K-means clustering. The results presented in Figure 4 use the outputs of Algorithms 1 and 2, respectively. This clustering method allows for the identification of high-density traffic zones, which are critical in detecting potential congestion. Moreover, when a cluster exceeds a predefined vehicle threshold, indicating congestion, the system triggers several actions: adjusting traffic signal timings, rerouting vehicles, or notifying nearby vehicles and emergency services. Additionally, the system can simulate random incidents, such as accidents, where traffic is automatically diverted, and signal timings are adjusted to manage the flow in affected areas.

## 5. Conclusions and Discussion

In this article, the ability of AI, particularly the K-means clustering algorithm, to address the challenges encountered by VANETs is examined. The application of K-means clustering serves to augment both the efficiency and reliability of communications by means of clustering methodologies, thereby enhancing the exchange of information among vehicles. The proposed integrated ITS framework, which leverages both VANETs and AI techniques, aspires to amplify data throughput while simultaneously diminishing response times, ultimately fostering enhanced safety features. Furthermore, this initiative aligns with the 2030 objectives established by the United Nations to reduce the mortality rate resulting from road accidents, which will improve healthcare, signifying that substantial advancements have been achieved thus far. In addition, AI-driven solutions pertaining to VANETs continue to proliferate, with vehicle communication and security enhancements being of paramount concern during this critical global juncture.

Furthermore, the methodology that has been put forth is heavily dependent on the establishment of V2V communication, which necessitates that each and every vehicle involved in this intricate system effectively communicates with all other vehicles, thereby fostering a collaborative network among them. To achieve such seamless communication, it becomes imperative for all vehicles that are part of the ITS framework to be equipped with specialized technological apparatus, which consequently leads to a significant escalation in the overall financial expenditure associated with the implementation of this system. This financial burden can be alleviated through the introduction of an incentive-based mechanism that could be initiated by insurance providers, a strategy that not only serves to enhance the understanding of driver behavior patterns but also provides a pathway for drivers to potentially reduce their insurance claims, thus creating a mutually beneficial scenario for both insurance companies and consumers alike. Conversely, the issue of congestion control emerges as a critical challenge when the volume of vehicles on the road reaches a point of significant density; in such scenarios, the high data rate requirements associated with effective communication necessitate the exploration of innovative and creative approaches to effectively manage and mitigate these issues. Consequently, conducting research that is centered on the potential utilization of higher-frequency ranges, specifically those that encompass millimeter wave (mmWave) and sub-terahertz (sub-THz) frequencies, could prove to be immensely beneficial as it may significantly enhance the capability to address and overcome the aforementioned challenges associated with congestion and communication in a densely populated vehicular environment.

## Figures and Tables

**Table 1:** Summary of existing literature on road traffic safety, VANETs, and AI applications with identified research gaps.

Reference	Study	Key Contributions	Literature Gap
[1]	Aziz et al. (2022)	Highlights road traffic accidents as a leading cause of death among young adults and children. Discusses the economic burden on low and middle-income countries.	The study does not propose specific technological solutions to address these fatalities and lacks integration with advanced communication systems.
[2]	Fondze nyuy et al. (2024)	Emphasizes the economic impact of road traffic accidents on national GDP (about 3%).	No focus on innovative approaches to mitigate fatalities using IoT or AI technologies.
[3]	Jacobs et al. (2000)	Discusses the UN goal of reducing road traffic deaths by 50% by 2030.	Does not elaborate on how technological advancements in communication or AI can contribute to this goal.
[5,6,7]	Li et al. (2015); Jamshed et al. (2020, 2022)	Introduces the integration of wireless communication and sensors for data acquisition, with a focus on long-distance communication.	Limited exploration of how these technologies can be applied to VANET and ITS for road safety.
[10,11]	Shafi et al. (2017); Nauman et al. (2019)	Discusses the role of 5G, mMTC, and URLLC in enhancing IoT device connectivity and reliability.	Limited focus on vehicle communication networks and safety improvements via 5G advancements.
[14]	Mchergui et al. (2022)	Reviews V2X communication, especially New Radio V2X (NR-V2X) and Sidelink communication, allowing direct vehicle-to-device communication.	Insufficient discussion of AI applications for optimizing V2X communication and scalability in VANETs.
[16]	Ayaz et al. (2023)	Details advances in VANET communication, addressing safety, commercial, and infotainment services.	Does not propose AI-based frameworks to further enhance VANET communication reliability and efficiency.
[17,18]	Hamdi et al. (2020); Mohammed et al. (2024)	Discusses VANETs as a type of MANET, highlighting their importance in Intelligent Transportation Systems (ITSs).	Lacks a structured AI approach to optimize communication in VANETs.
[13,20,28]	Jamshed et al. (2021, 2024); Shrestha et al. (2018)	Recognizes AI’s potential (ML, DL, and SI) in solving VANET challenges, with K-means clustering showing promise for data exchange in VANETs.	Limited exploration of scalable, AI-based VANET frameworks designed for practical ITS deployment.
N/A	This paper (2024)	Proposes a multi-layered VANET-enabled ITS framework using K-means clustering to improve scalability and reliability, contributing towards the UN goal of reducing road fatalities by 50%.	Addresses the gap by introducing a novel AI-driven VANET framework, optimized for communication and safety in ITS.

## Data Availability

Data are contained within the article.
